# Unveiling the shield: Troglitazone's impact on epilepsy‐induced nerve injury through ferroptosis inhibition

**DOI:** 10.1111/cns.14911

**Published:** 2024-08-15

**Authors:** Zhi‐Bin Wang, Jun‐Yan Liu, Shi‐Long Jiang, Wei Zhuo, Pan Xie, Wen‐Ting Dai, Xiao‐Yuan Mao, Zhao‐Qian Liu

**Affiliations:** ^1^ Department of Clinical Pharmacology, Hunan Key Laboratory of Pharmacogenetics, National Clinical Research Center for Geriatric Disorders, Xiangya Hospital Central South University Changsha P.R. China; ^2^ Hunan Cancer Hospital, The Affiliated Cancer Hospital of Xiangya School of Medicine Central South University Changsha P.R. China; ^3^ Department of Orthopaedics, Xiangya Hospital Central South University Changsha P.R. China; ^4^ Institute of Clinical Pharmacology, Engineering Research Center for Applied Technology of Pharmacogenomics of Ministry of Education Central South University Changsha P.R. China

**Keywords:** brain damage, epilepsy, ferroptosis, neuroprotection, Plaur, troglitazone

## Abstract

**Background:**

Epilepsy is a widespread central nervous system disorder with an estimated 50 million people affected globally. It is characterized by a bimodal incidence peak among infants and the elderly and is influenced by a variety of risk factors, including a significant genetic component. Despite the use of anti‐epileptic drugs (AEDs), drug‐refractory epilepsy develops in about one‐third of patients, highlighting the need for alternative therapeutic approaches.

**Aims:**

The primary aim of this study was to evaluate the neuroprotective effects of troglitazone (TGZ) in epilepsy and to explore the potential mechanisms underlying its action.

**Methods:**

We employed both in vitro and in vivo models to assess TGZ's effects. The in vitro model involved glutamate‐induced toxicity in HT22 mouse hippocampal neurons, while the in vivo model used kainic acid (KA) to induce epilepsy in mice. A range of methods, including Hoechst/PI staining, CCK‐8 assay, flow cytometry, RT‐PCR analysis, Nissl staining, scanning electron microscopy, and RNA sequencing, were utilized to assess various parameters such as cellular damage, viability, lipid‐ROS levels, mitochondrial membrane potential, mRNA expression, seizure grade, and mitochondrial morphology.

**Results:**

Our results indicate that TGZ, at doses of 5 or 20 mg/kg/day, significantly reduces KA‐induced seizures and neuronal damage in mice by inhibiting the process of ferroptosis. Furthermore, TGZ was found to prevent changes in mitochondrial morphology. In the glutamate‐induced HT22 cell damage model, 2.5 μM TGZ effectively suppressed neuronal ferroptosis, as shown by a reduction in lipid‐ROS accumulation, a decrease in mitochondrial membrane potential, and an increase in PTGS2 expression. The anti‐ferroptotic effect of TGZ was confirmed in an erastin‐induced HT22 cell damage model as well. Additionally, TGZ reversed the upregulation of Plaur expression in HT22 cells treated with glutamate or erastin. The downregulation of Plaur expression was found to alleviate seizures and reduce neuronal damage in the mouse hippocampus.

**Conclusion:**

This study demonstrates that troglitazone has significant therapeutic potential in the treatment of epilepsy by reducing epileptic seizures and the associated brain damage through the inhibition of neuronal ferroptosis. The downregulation of Plaur expression plays a crucial role in TGZ's anti‐ferroptotic effect, offering a promising avenue for the development of new epilepsy treatments.

## Graphical abstract

Troglitazone can provide significant neuroprotection against neuronal damage. Troglitazone demonstrates remarkable therapeutic efficacy in alleviating seizure occurrence in mice with an epileptic model. The neuroprotective action of troglitazone is mediated through the inhibition of Plaur, and downregulation of Plaur can mimic this protective effect. Drawn by Figdraw (www.figdraw.com)

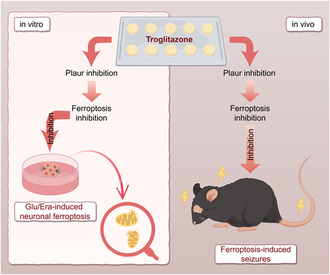

## 1 INTRODUCTION

Epilepsy, a chronic neurological disorder caused by aberrant synchronization of neuronal discharge in the brain occurs from adolescence to old age. It is estimated that nearly 70 million people suffer from epilepsy worldwide and there are about 10 million people with epilepsy in China.^1^, ^2^, ^3^ Pharmacotherapy is the mainstay to control seizures. However, there are still estimated one‐third of cases who are refractory to current medications, namely drug‐resistant epilepsy.^4^, ^5^ It is of urgent need to develop new drugs and explore the potential molecular mechanism.

Generally, seizures result from an imbalance in the levels of excitatory and inhibitory neurotransmitters. The disrupted equilibrium is characterized by an increase in excitatory neurotransmitter glutamate and a decrease in inhibitory neurotransmitter gamma‐aminobutyric acid (GABA).^6^, ^7^ These alterations ultimately lead to the accumulation of reactive oxygen species (ROS), neurotoxicity, neuronal cell death, and the onset of epilepsy.^8^, ^9^ Recent investigations have demonstrated that inhibiting the production of ROS or reactive nitrogen species (RNS) can improve survival rates, alleviate cognitive impairments, and reduce neuronal damage in cases of persistent epilepsy. Encouraging anti‐epileptic effects have also been achieved during the development of epilepsy in rats with chronic kainic acid (KA)‐induced epilepsy, employing a newly developed antioxidant that readily crosses the blood–brain barrier.^10^, ^11^

Ferroptosis, a novel form of regulated cell death introduced by Professor Stockwell in 2012, stands apart from apoptosis and autophagy.^12^ Its key features include iron‐dependent accumulation of lethal lipid ROS. Morphologically, ferroptosis is typified by disrupted plasma membrane integrity, cytoplasmic swelling, organelle enlargement, and moderate chromatin condensation. Ultrastructurally, ferroptosis often presents with mitochondrial aberrations such as clustering or expansion, heightened membrane density, decreased or absent cristae, and outer membrane rupture.^13^ Importantly, mitochondria serve as pivotal sites for ROS generation in cells. Numerous investigations have indicated that it is specifically the ROS produced by mitochondria that contribute to the initiation of ferroptosis.^14^, ^15^, ^16^, ^17^

Emerging evidence suggests the involvement of ferroptosis in diverse biological processes, encompassing cancer, neurodegeneration, tissue injury, inflammation, and infection.^15^, ^18^, ^19^ Inducers of ferroptosis, such as erastin and RSL3, have displayed tumor growth inhibition potential through the induction of ferroptosis.^20^, ^21^, ^22^ Interestingly, the ferroptosis inhibitor SRS11‐92 has demonstrated the restoration of healthy neurons and protection of oligodendrocytes against cysteine deprivation‐induced neural damage in a Huntington's disease model. Notably, iron chelators like DFO have shown promise in preventing the progression of Alzheimer's and Parkinson's diseases by enhancing hypoxia‐inducible factor‐1 (HIF‐1) stability in the brain and inhibiting neuronal death.^1^ This suggests that ferroptosis plays an important role in the occurrence of neurological diseases,^23^, ^24^ and inhibition of ferroptosis exerts neuroprotective or therapeutic effects. Our previous studies have confirmed that ferroptosis indeed occur during the onset of epilepsy and pharmacological inhibition of ferroptosis process by specific inhibitors including ferrostatin‐1 and liproxstatin‐1 significantly alleviates epileptic seizures.^25^, ^26^ Therefore, intervention of ferroptosis process may be helpful for epilepsy treatment.

In 2017, investigators discovered that thiazolidinediones, a well‐established antihyperglycemic medication, exhibited potent inhibitory effects on RSL3‐induced ferroptosis in mouse embryonic fibroblasts.^27^ Thiazolidinediones are widely acknowledged for their ability to activate peroxisome proliferator‐activated receptor‐γ (PPAR‐γ) within the nucleus, thereby enhancing insulin sensitivity, promoting glucose metabolism, and ultimately reducing blood sugar levels. This is the classical mechanism of action of thiazolidinediones.^28^, ^29^ Common thiazolidinediones include troglitazone (TGZ), rosiglitazone, and pioglitazone. TGZ has been limited in clinical practice due to its serious hepatotoxicity. Rosiglitazone increases the risk of cardiovascular diseases and raises LDL‐C and triglyceride levels. Pioglitazone has a more comprehensive effect on blood sugar reduction and lipid regulation, improves cardiovascular outcomes, and partially activates the PPAR‐α receptor. It is worth noting that compared with rosiglitazone and pioglitazone, TGZ has the strongest inhibitory effect on ferroptosis. Therefore, TGZ is selected for research purpose.^27^

It has been reported that rosiglitazone, the similar structure to TGZ, possesses seizure‐suppressing effect in epileptic animal models; however, the concrete mechanism remains uncharacterized.^30^ Distinguishing itself from previous studies, our present research endeavor aims to unveil the key targets of neuroprotective effects exerted by TGZ through RNA sequencing. Additionally, we employed AAV viral vectors to achieve specific inhibition of these targets in the hippocampal region in vivo, thus validating their biological functionality. This study's innovation lies in two aspects: firstly, the repurposing of an existing medication, demonstrating the safe and effective use of an “old drug” as an anti‐epileptic agent; secondly, the elucidation of the specific mechanism underlying TGZ's neuroprotective effects by identifying critical target sites.

Herein, the present work was conducted to assess the neuroprotective effect of TGZ against epileptic seizure and explore the potential mechanism underlying its neuroprotection. We found that TGZ alleviated epileptic seizures via suppressing neuronal ferroptosis and downregulation of Plaur expression was involved in the anti‐ferroptotic effect of TGZ. Our data suggest that TGZ serves as a promising drug for combating seizures.

## 2 MATERIALS AND METHODS

### 2.1 Drugs and reagents

TGZ (S8432) and erastin (S7242) were obtained from Selleck Chemicals. Glutamate (G8415) and kainic acid (K0250) was were purchased from Sigma‐Aldrich. Hoechst 33342/PI (ST511) was purchased from Beyotime Biotechnology. BODIPYTM 581/591 C11 (D3861) was purchased supplied from by Invitrogen. Mitochondrial Membrane Potential Assay Kit with JC‐1 (M8650) was purchased from Solarbio. AAV‐Plaur and AAV‐control were constructed by Shanghai Genechem.

### 2.2 Experimental animals

Male C57BL/6J mice aged 6 to 8 weeks were provided by Central South University's Center for Animals. All rats were individually housed at a constant temperature of 22 ± 2°C, with lights turned out at 20:00 and a cycle of 12 h of light and 12 h of darkness. All animal experiments were conducted in strict accordance with principles presented in the National Institute of Health for the Care and Use of Laboratory Animals and approved by the Institutional Animal Ethics Committee of Central South University (approved number: 2017–1103).

**FIGURE 1:**
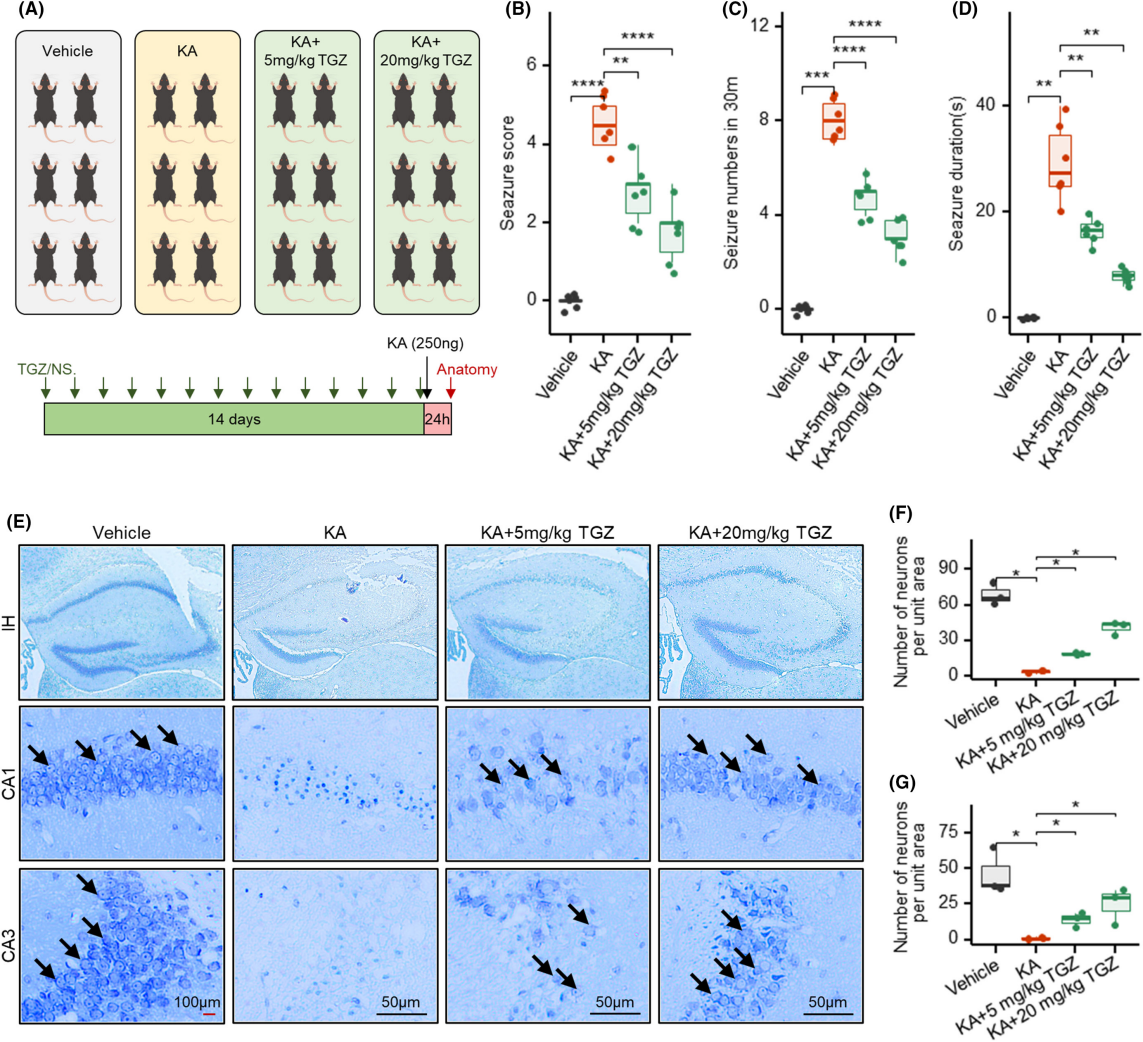
Troglitazone improves KA‐induced seizures and neuronal damage. (A) Schematic diagram of the experimental design. (B‐D) Effects of TGZ on the seizure score, seizure duration, and seizure numbers (in 30 m) 24 h after KA injection in different groups. (E) The illustration of Nissl staining in several groups' CA1 and CA3 hippocampus subregions 24 h after KA injection. Arrows indicate Nissl‐positive cells. Red scale bar indicates 100 μM; black scale bar indicates 50 μM. (F, G) Statistical analysis of viable neurons in CA1 and CA3 regions 24 h after KA injection in different groups. *p < 0.05; **,*p* < 0.01; ***p < 0.001; ***p < 0.0001. IH, surgical ipsilateral hippocampus; KA, kainic acid; NS, normal saline; TGZ, troglitazone. Shapiro–Wilk test is applied for normality checks. *T*‐test is used for two groups or ANOVA for multiple groups if data are normal; otherwise, we used Wilcoxon test for non‐normal data.

Mice were divided into four following groups: the control group (*n* = 6), the epilepsy model group, and TGZ‐treated groups (the dose of TGZ setting at 5 and 20 mg/kg/day, respectively). The control and epilepsy model groups received an equivalent dose of saline solution. After the completion of the epilepsy model, the treatment was initiated, and the medication was administered by gavage for 14 consecutive days. Subsequently, various indicators of epileptic seizures were measured, and samples were collected for biochemical analysis (Figure 1A). In the preliminary experiments, both intraperitoneal injection and gavage administration were attempted. It was found that gavage administration was more effective, and clinically, troglitazone is also administered orally. Therefore, this study chose gavage as the method of drug administration. Additionally, the dosage was set with a concentration gradient to better explore the pharmacological effects of TGZ.

As described in our previous study, 250 ng/μL KA, which induces seizures, was injected into hippocampus of mice to induce an epilepsy model.^31^ Mice in vehicle group were injected with an equal volume of normal saline in the same area of the brain. In experiments exploring the effects of knockdown *Plaur* on seizures in mice, mice were divided into three groups. Adeno‐associated virus (AAV) or control reagents were injected in the hippocampal region of the mouse brain 28 d prior to epileptic molding (Figure 6A). KA and AAV were injected at a location 2.0 mm posterior to the anterior fontanelle and 1.8 mm to the right, with a depth of 2.3 mm. 1 μL of AAV is slowly injected over 5 min (0.2 μL per minute). After the injection is complete, it is necessary to wait for 5 min to allow the drug to fully diffuse. Then, the needle is slowly withdrawn.

### 2.3 Seizure behavior evaluation

According to the Racine's scale,^32^ seizure behavior was evaluated. The following description elaborated on the standards of Racine stages: Stages include Stage 0—no response; Stage 1—rhythmic twitching of the face and whiskers; Stage 2—head bobbing and circling; Stage 3—myoclonic and limb spasms; Stage 4—rearing and falling; Stage 5—general tonic–clonic seizures with running and jumping; and Stage 6—death. Mice that reached the third seizure stage or above were deemed to have been effectively kindled and were recruited in the ensuing experiment.

### 2.4 Nissl staining

The Nissl stain solution was used to find Nissl body, which is a sign of a normal neuron. Nissl staining solution was briefly applied to brain tissue sections (5 μM) from each group and allowed to sit at room temperature for 10 min. The brain slides were dehydrated before being cover‐slipped with neutral balsam and photographed using a light microscope. Image J was used to compute the Nissl bodies stained by the Nissl solution in the hippocampus CA1 and CA3 subregions.

### 2.5 Detection of mitochondrial membrane potential by JC‐1 staining

Dissolve 50 μL JC‐1 (200×) in 8 mL pure water, shake vigorously and mix well, then add 2 mL buffer (5×) to make a working solution. After discard the medium and washing the cells with PBS, add 250 μL of medium and 250 μL of working solution, mix thoroughly and incubate at 37°C for 20 min. Add 1 mL of JC‐1 buffer (5×) to 4 mL of distilled water to make the wash solution and set aside in an ice bath. When the incubation is complete, the cells are washed twice using the wash solution. After adding 500 μL of PBS, the assay was performed using flow cytometry.

### 2.6 Lipid‐ROS

C11‐BODIPY was used to detect the oxidation of lipids (Invitrogen). After various treatments, cells were collected and then treated with 2 μM C11‐BODIPY at 37°C for 20 min. The cells were then washed twice with PBS. Using flow cytometry, the level of Lipid‐ROS within cells was assessed.

### 2.7 Cell culture

HT22, an immortalized hippocampal cell line obtained from mice, was cultured in Dulbecco's modified Eagle's medium with high glucose, 10% fetal bovine serum (FBS), and 1% penicillin/streptomycin in an incubator at 37°C with 5% CO_2_.

### 2.8 RNA sequencing

RNA sequencing was conducted by Novogene Co., Ltd. (Tianjin, China) after treatments. With the Illumina TruSeq RNA Sample Prep Kit, libraries were constructed using purified RNA from five groups, including control, glutamate group, glutamate+TGZ group, erastin group, and erastin+TGZ group. The quality of the created library was evaluated using an Agilent 2100 Bioanalyzer. Cufflinks was used to calculate the fragments per kilobase per million reads (FPKM) value of each gene after htseq‐count was used to gather the read counts of each gene.

As a criterion for determining the importance of differentially expressed genes (DEGs), a *p*‐value of less than 0.05 and a fold‐change of greater than 2 were established as the threshold.

### 2.9 Quantitative PCR

Total RNA was isolated from HT22 cells or hippocampus tissue using RNAiso Plus (TaKaRa, #9109, Japan). Following the manufacturer's instructions, 1 μg of RNA was reverse‐transcribed into cDNA using a PrimeScript RT Reagent Kit with gDNA Eraser (TaKaRa, #RR047A, Japan). On a LightCycler@480II/96 (Roche, Switzerland) equipped with a SYBR Premix Dimer Eraser Kit (Takara, #RR091A, Japan), qRT‐PCR was conducted. ACTB was utilized to standardize the levels of expression. For statistical analysis and determination of *p* values, the set of △Ct replicates was utilized. The expressions of mRNA from each group were determined using 2^−△△Ct^ method. The primers were provided by BioSune Biotechnology.

### 2.10 Western blot assay

Hippocampal tissues or cells were lysed in a cold lysis solution (P0013, Beyotime Biotechnology Institute, China) containing a mixture of protease and phosphatase inhibitors to extract total proteins. 20 g of proteins was run on a gel electrophoresis system, transferred to polyvinylidene difluoride (PVDF) membrane, and then blocked with five percent non‐fat milk for 1 h at room temperature. After that, we incubated the membranes with primary antibodies such as PLAUR (with ACTB serving as a control). The protein bands were then detected using an enhanced chemiluminescence kit after being probed with horseradish peroxidase (HRP) conjugated secondary anti‐mouse or anti‐rabbit antibodies for 1 h the following day. The distinction in protein expression was analyzed using the imageJ.

### 2.11 Statistical analysis

Means and standard deviations of the data were plotted (SEM). Normality tests were conducted on each group of data using the Shapiro–Wilk test. If the data meet the assumption of normal distribution, we performed difference analysis using *t*‐test for two groups of data or ANOVA for three or more groups of data. If the normal distribution is not met, a non‐parametric method (Wilcoxon test) was employed. The significance level for the data was set at *p <* 0.05.

## 3 RESULTS

### 3.1 Troglitazone improves KA‐induced seizures and neuronal damage

To investigate the effect of TGZ on KA‐induced seizures in mice, we scored the seizures after administration of KA (Figure 1A). The results showed that for the vehicle group, the KA drug significantly induced seizure onset in mice with a seizure class greater than Grade 4, a single duration greater than 8 seconds and a number of seizures greater than 20 in 30 min in the KA group (Figure 1B–D). Both 5 and 20 mg/kg of TGZ significantly suppressed KA‐induced seizures in mice (Figure 1B). The mice in the troglitazone group had a seizure class of less than 2. The duration of a single seizure in group of TGZ administration was about 10 s, and the number of seizures in 30 min was less than 6 (Figure 1C,D).

By performing Nissl staining on the hippocampal tissues of each group of mice, we found that a significant reduction of neuronal cells was observed in the CA1 region and CA3 region of the hippocampal tissues of mice in the KA‐induced group, compared with the control group. In contrast, TGZ significantly improved neuronal survival in KA mouse model (Figure 1E–G).

### 3.2 Ferroptosis is involved in the neuroprotection of troglitazone in KA‐induced epilepsy mouse

**FIGURE 2:**
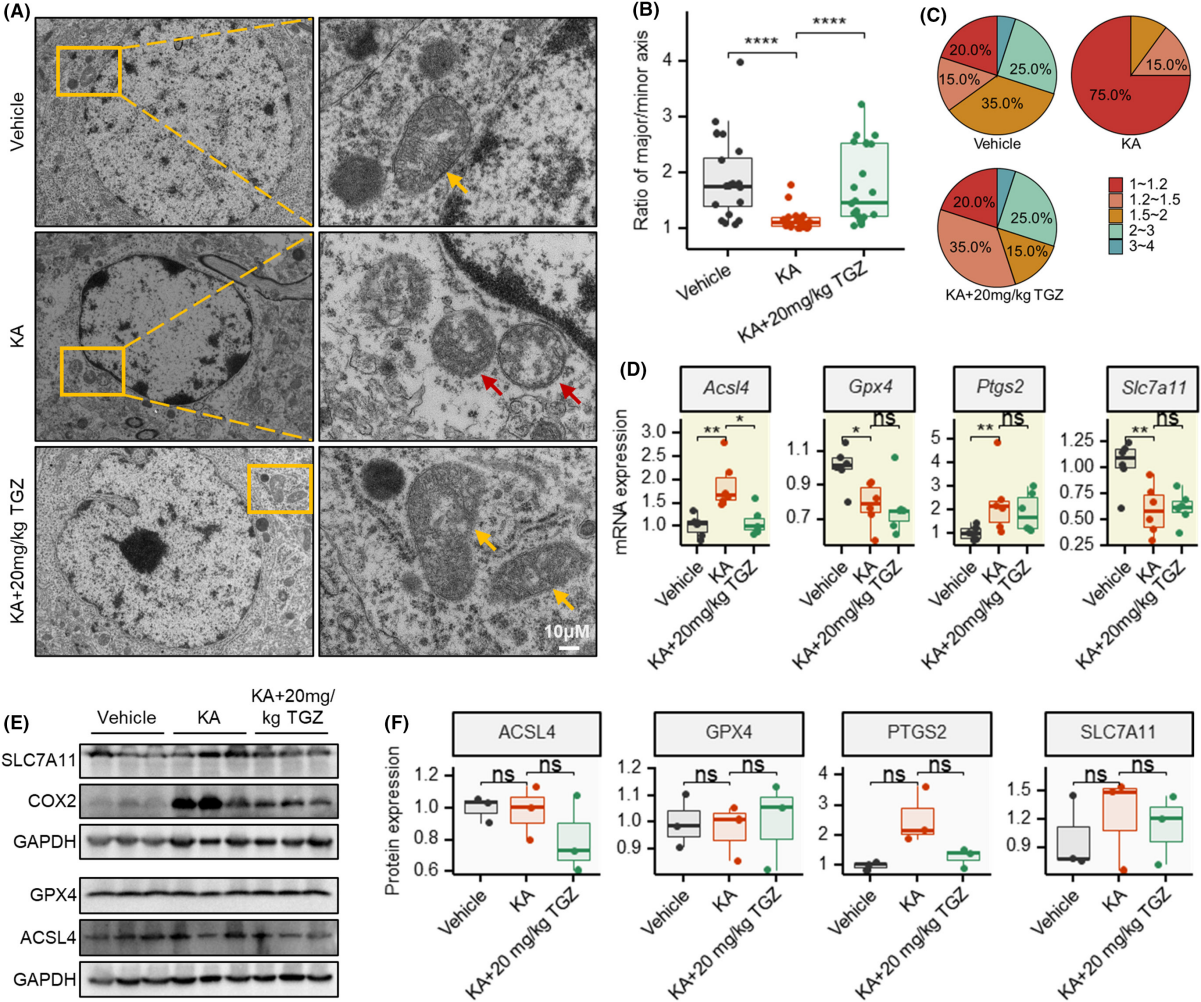
Ferroptosis is involved in the neuroprotection of troglitazone in KA‐induced epilepsy mouse. (A) Transmission electron micrograph of mitochondria in mouse right hippocampal cells 24 h after KA injection in different groups. (B, C) Statistical results for the ratio and percentage of mitochondrial major to miner axis. (D) MRNA expression of ferroptosis‐related genes Acsl4, Gpx4, Ptgs2 (encoding protein COX2), and Slc7a11. (E, F) Expression of ferroptosis‐related proteins. *p < 0.05; **p < 0.01; ***p < 0.001; ***p < 0.0001. KA, kainic acid; TGZ, troglitazone. Shapiro–Wilk test is applied for normality checks. T‐test is used for two groups or ANOVA for multiple groups if data are normal; otherwise, we used Wilcoxon test for non‐normal data.

To further investigate what type of neuronal damage KA induces and how TGZ exerted its protective effect, we observed morphological changes in hippocampal cells using electron microscopy. We found that neuronal cells in the hippocampus of the KA‐induced group of mice showed reduced size, crumpled mitochondrial membranes, and fragmented and disordered cristae, and that these changes were a distinctive feature of ferroptosis (Figure 2A). Interestingly, we found that TGZ significantly inhibited the mitochondrial changes in neuronal cells induced by KA. According to the statistical results, neuronal cells in the KA‐induced group were generally wrinkled and approached a round ball shape (the ratio of major/minor axis was close to 1), whereas the mitochondrial morphology of neurons could be well restored in TGZ‐treated group (Figure 2B,C).

We further examined *Acsl4*, *Gpx4*, *Ptgs2*, and *Slc7a11*, which are ferroptosis‐related indicators. The results showed that KA significantly downregulated the expression profiles of *Gpx4* and *Slc7a11*, but TGZ treatment did not significantly restore the decreased expression levels of *Gpx4* and *Slc7a11* (Figure 2D). It suggests that the protection of neuronal cells by TGZ may not act through the classical ferroptosis regulatory genes such as *Gpx4* and *Slc7a11*. Furthermore, in Figure 2E,F, it was observed that the expression level of COX2 (the encoded protein of *Ptgs2*) was significantly upregulated in the KA group compared with the control group (*p* = 0.028). However, the KA + TGZ group was able to partially reduce the KA‐induced upregulation of COX2 (with an inhibitory trend but without statistical significance).

### 3.3 Troglitazone significantly inhibits neuronal ferroptosis in glutamate‐induced cell death in mouse hippocampal HT22 cells

**FIGURE 3:**
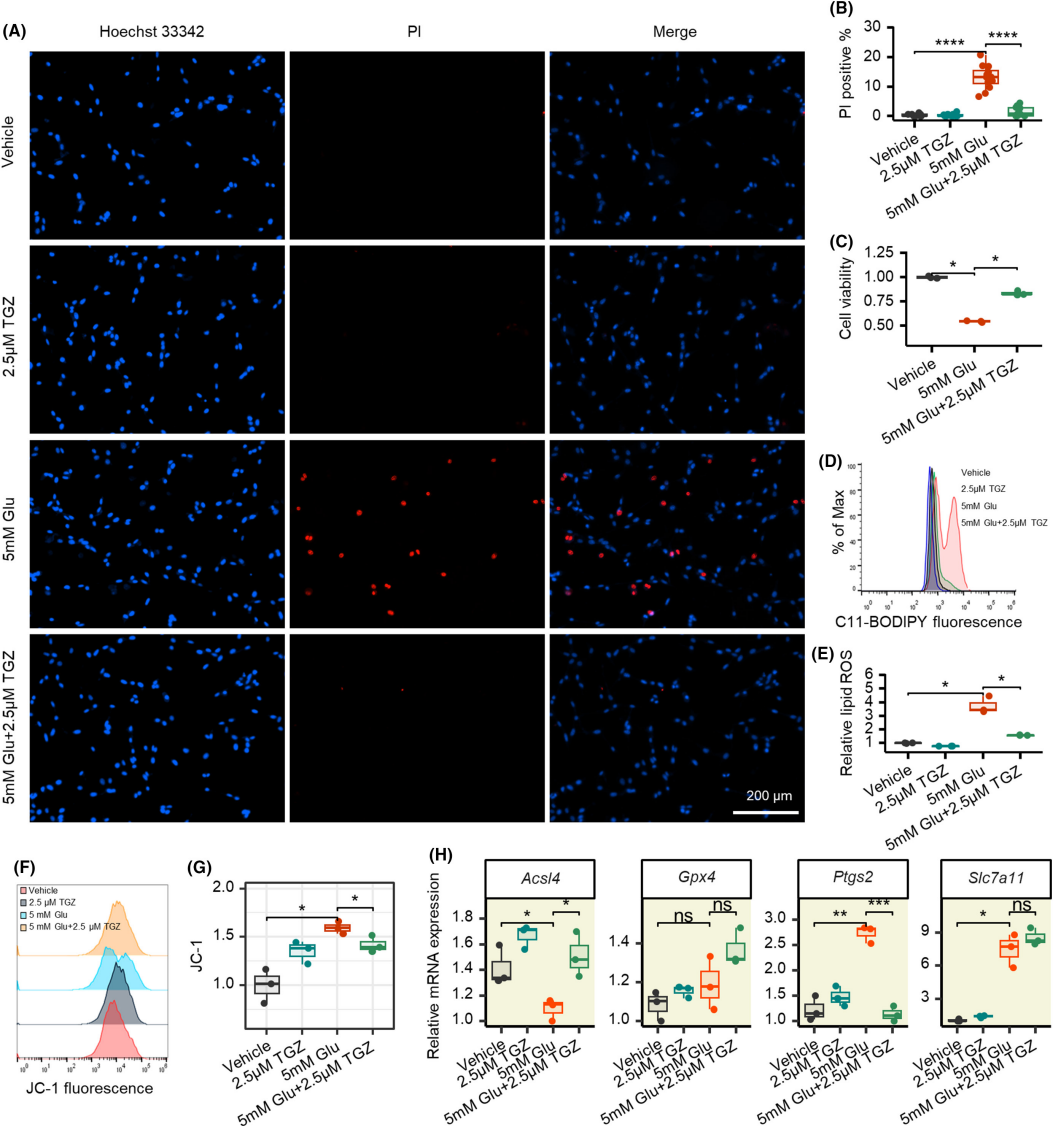
Troglitazone significantly inhibits neuronal ferroptosis in glutamate‐induced cell death in mouse hippocampal HT22 cells. (A) Representative images of Hoechst 33342/PI staining 8 h after treated with 5 mM glutamate or 2.5 μM troglitazone in HT22 cells. (B) Statistical analysis of Hoechst 33342/PI staining results in different groups. (C) Measurement of cell viability upon 5 mM glutamate with or without 2.5 μM troglitazone with CCK8. (D) Lipid ROS production assessed 8 h after treated with 5 mM glutamate or 2.5 μM troglitazone in HT22 cells by flow cytometry using C11‐BODIPY. (E) Statistical analysis of flow cytometry results in different groups. (F) Change in mitochondrial membrane potential in different groups detected by JC‐1 fluorescent probe and examined by flow cytometry. (G) Statistical analysis mitochondrial membrane potential results in different groups. (H) MRNA expression of ferroptosis‐related genes Acsl4, Gpx4, Ptgs2, and Slc7a11. *p < 0.05; **p < 0.01; ***p < 0.001; ***p < 0.0001. Glu, glutamate; TGZ, troglitazone. Shapiro–Wilk test is applied for normality checks. T‐test is used for two groups or ANOVA for multiple groups if data are normal; otherwise, we used Wilcoxon test for non‐normal data.

We further validated the neuroprotective effect of TGZ in vitro. The results showed that TGZ significantly inhibited neuronal cell damage in HT22 following glutamate stimulation (Figure 3A,B).

CCK8 cell viability assays, similarly, illustrated the neuroprotective effect of troglitazone (Figure 3C). In particular, we found that TGZ remarkably inhibited the accumulation of Lipid‐ROS induced by glutamate (Figure 3D,E).

One of the distinctive features of ferroptosis is the alteration of mitochondrial morphology and function. To assess the changes in mitochondrial membrane function, we examined the alteration of mitochondrial membrane potential using the JC‐1 probe. The results showed that the mitochondrial membrane potential of cells in the glutamate‐treated group was significantly reduced (lower JC‐1 fluorescence intensity) compared with the control group, and that TGZ inhibited the altered membrane potential well (Figure 3F,G). In addition, we noted that *Ptgs2*, a downstream marker of ferroptosis, was significantly elevated in both the glutamate‐treated groups, whereas TGZ could downregulate the elevated *Ptgs2* expression levels (Figure 3H). This again confirms the inhibitory effect of troglitazone on ferroptosis.

### 3.4 Troglitazone was further shown to inhibit ferroptosis in Erastin‐induced neuronal ferroptosis model

**FIGURE 4:**
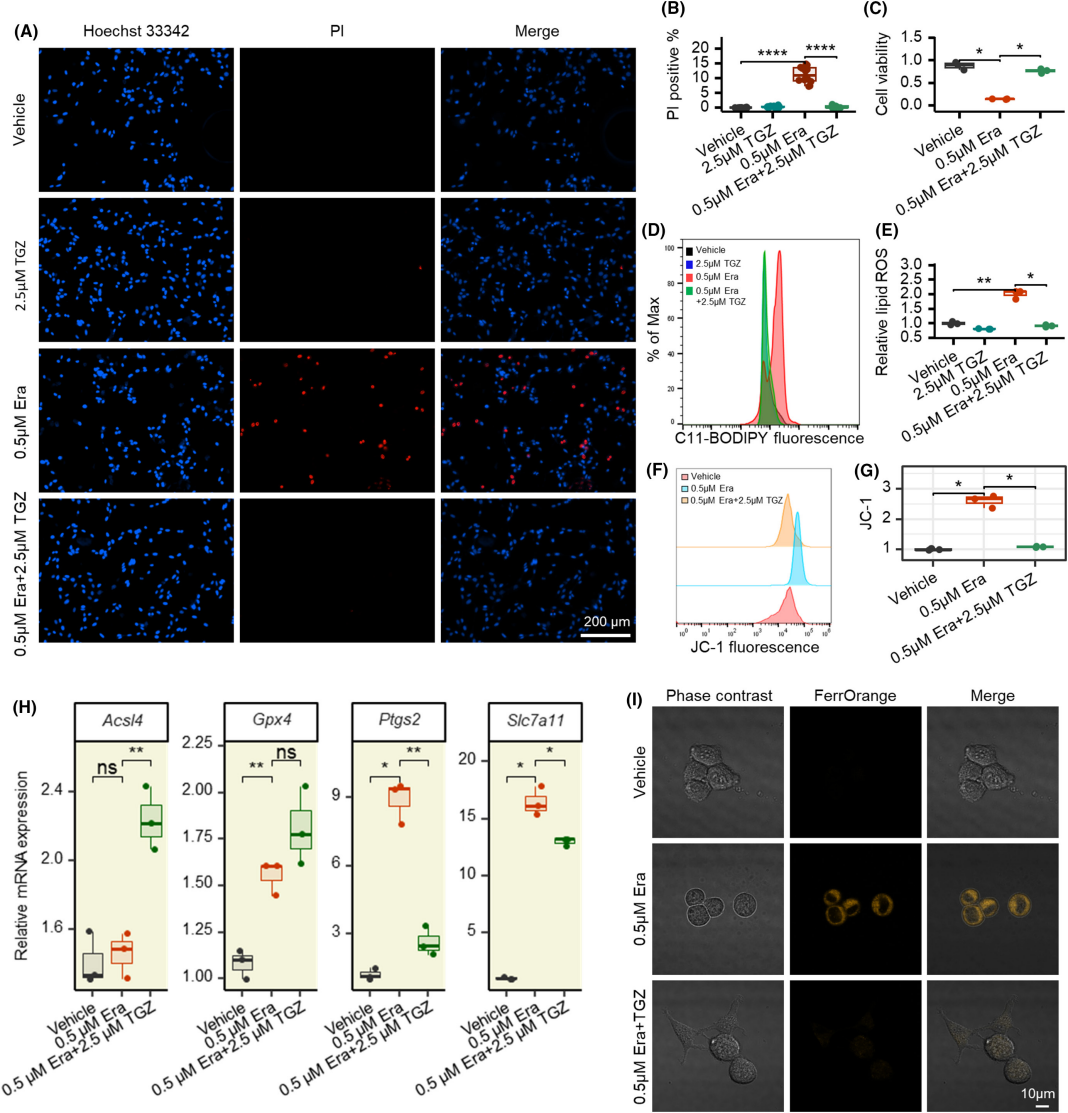
Troglitazone was further shown to inhibit ferroptosis in erastin‐induced neuronal ferroptosis model. (A) Representative images of Hoechst 33342/PI staining 8 h after treated with 0.5 μM erastin or 2.5 μM troglitazone in HT22 cells. (B) Statistical analysis of Hoechst 33342/PI staining results in different groups. (C) Measurement of cell viability upon 0.5 μM erastin with or without 2.5 μM troglitazone with CCK8. (D) Lipid ROS production assessed 8 h after treated with 0.5 μM erastin or 2.5 μM troglitazone in HT22 cells by flow cytometry using C11‐BODIPY. (E) Statistical analysis of lipid ROS results in different groups. (F) Change in mitochondrial membrane potential in different groups detected by JC‐1 fluorescent probe and examined by flow cytometry. (G) Statistical analysis mitochondrial membrane potential results in different groups. (H) MRNA expression of ferroptosis‐related genes Acsl4, Gpx4, Ptgs2, and Slc7a11. (I) Laser confocal microscopy images is used to detect the content of ferrous ions. *p < 0.05; **p < 0.01; ***p < 0.001; ***p < 0.0001. Glu, glutamate; TGZ, troglitazone. Shapiro–Wilk test is applied for normality checks. T‐test is used for two groups or ANOVA for multiple groups if data are normal; otherwise, we used Wilcoxon test for non‐normal data.

To further clarify the role of ferroptosis in the neuroprotective effects of TGZ, we explored the protective effects of TGZ using a ferroptotic cell model triggered by erastin. The results suggested that TGZ could dose‐dependently inhibit the ferroptosis cell injury induced by erastin (Figure 4A,B), and the CCK8 cell viability assay further confirmed the inhibitory effect of TGZ on ferroptosis (Figure 4C). Lipid‐ROS accumulation was significant high in the erastin‐treated cells compared with the control group, while TGZ could well inhibit the accumulation of Lipid‐ROS (Figure 4D,E). Furthermore, TGZ could also inhibit the decrease in mitochondrial membrane potential induced by erastin in HT22 neuronal cells (Figure 4F,G).

The expression level of *acsl4* was significantly decreased in the glutamate‐treated group, but not in the erastin‐treated group (Figures 3H and 4H), suggesting that changes in *acsl4* are not a common cause of the protective effect of TGZ in glutamate injury and erastin injury. Similarly, the expression level of *slc7a11* was elevated in both the glutamate‐treated group and the erastin‐treated group (Figures 3H and 4H). Considering that *slc7a11* is a suppressor gene of ferroptosis, the change in *slc7a11* here was a stressful change and again was not responsible for the neuroprotective effect of troglitazone.

### 3.5 Plaur as an important target of TGZ to inhibit neuronal ferroptosis

To further investigate the specific mechanism by which TGZ exerted its neuroprotective effect, we used RNA sequencing to observe the alteration of intracellular gene expression profiles by TGZ in the glutamate injury model and the erastin injury model, respectively.

**FIGURE 5:**
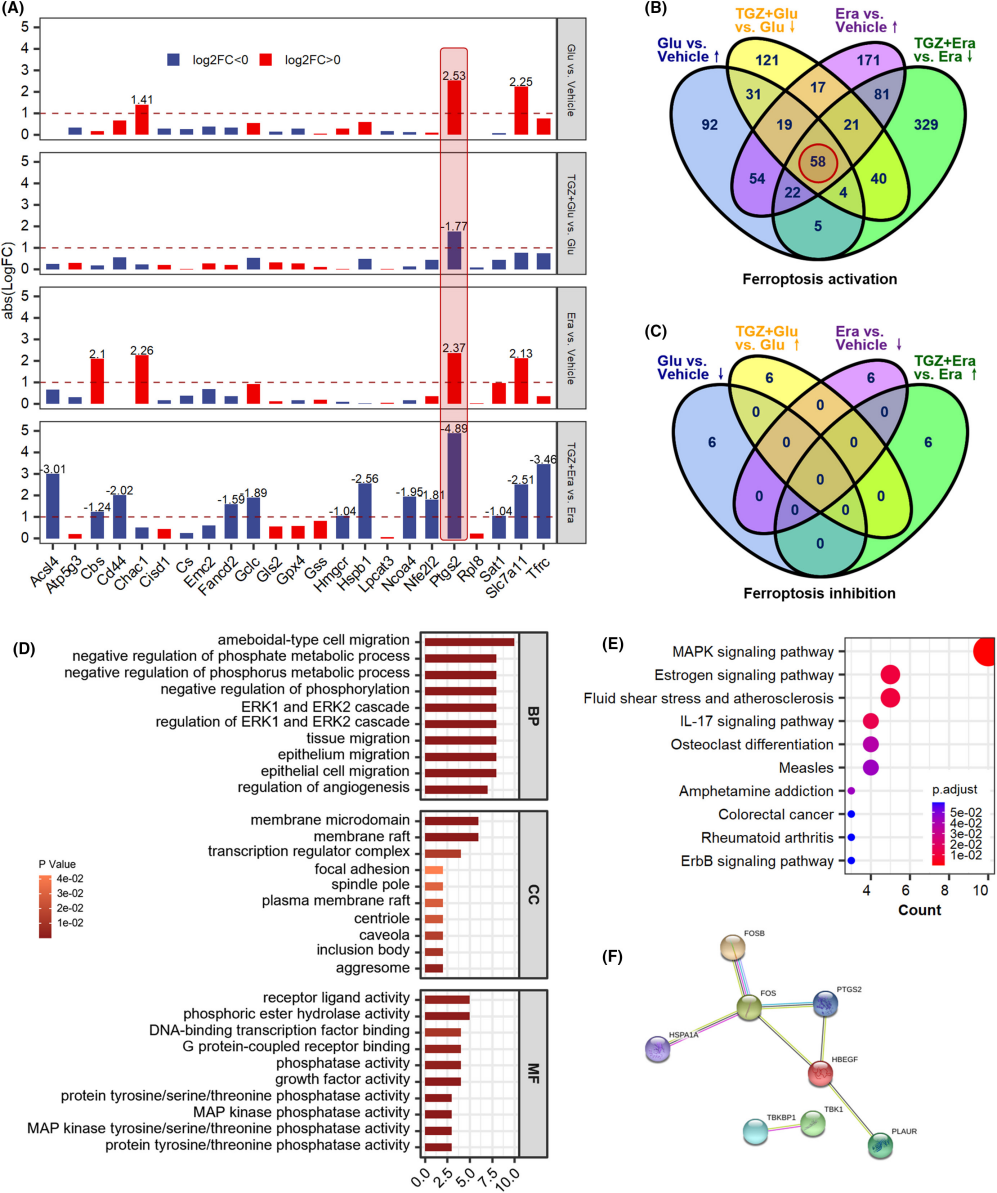
Plaur as an important target of troglitazone to inhibit neuronal ferroptosis. (A) MRNA expression of previously reported ferroptosis‐related genes. (B‐C) Veen diagram shows the number of ferroptosis activation or inhibition DEGs between different groups. (D) Gene ontology (GO) annotations of DEGs. (E) KEGG pathway enrichment for DEGs analyzed by clusterProfiler package. (F) Hub gene regulatory network mapped by String website. Glu, glutamate; Era, erastin; TGZ, troglitazone.

The sequencing results showed that, as with the previous cellular results, the ferroptosis marker *Ptgs2* was significantly upregulated in the glutamate‐treated group and the erastin‐treated group, and that TGZ inhibited this change (Figure 5A). We further searched for genes that were altered in both of the above cell injury models following TGZ intervention as potential regulators of the neuroprotective effects of TGZ (Figure 5B,C). A total of 58 genes that promote ferroptosis and may play a role in the neuroprotection of TGZ. Through GO enrichment analysis and pathway analysis, we found that these genes were significantly enriched in GO categories related to protein phosphorylation and membrane function, as well as in pathways such as the MAPK signaling pathway and the estrogen signaling pathway (Figure 5D,E). Using Gene Hub analysis, we found that Plaur is in a core regulatory position with Hbegf for these genes (Figure 5F).

### 3.6 Inhibition of Plaur suppresses seizures and neuronal damage in KA‐induced epilepsy mouse

**FIGURE 6:**
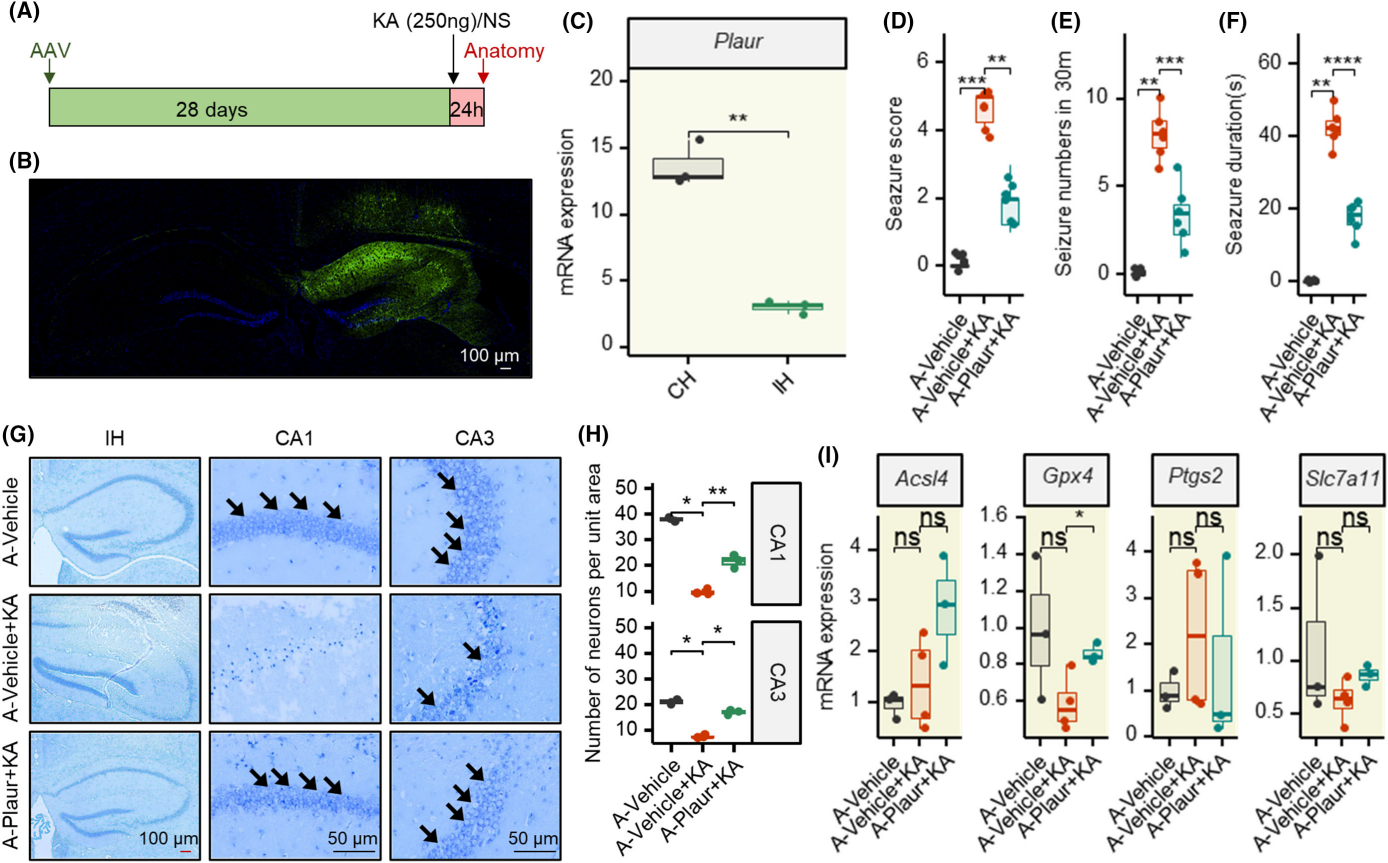
Inhibition of Plaur suppresses seizures and neuronal damage in KA‐induced epilepsy mouse. (A) Schematic diagram of the experimental design. (B) Representative brain showing AVV injection effect and resulting GFP distribution. Blue, DAPI; green, GFP. (C) MRNA expression levels of Plaur in the surgical ipsilateral hippocampus of mice 28 days after AAV‐Plaur injection (relative to the left hippocampus). (D‐F) Effects of AAV‐Plaur on the seizure score, seizure duration, and seizure numbers (in 30 m) 24 h after KA injection in different groups. (G) The illustration of Nissl staining in several groups' CA1 and CA3 hippocampus subregions 24 h after KA injection. Arrows indicate Nissl‐positive cells. Red scale bar indicates 100 μM; black scale bar indicates 50 μM. (H) Statistical analysis of viable neurons in CA1 and CA3 regions 24 h after KA injection in different groups. (I) MRNA expression of ferroptosis‐related genes Acsl4, Gpx4, Ptgs2, and Slc7a11. *p < 0.05; **p < 0.01; ***p < 0.001; ***p < 0.0001. AAV‐P, AAV‐Plaur; CH, surgical contralateral hippocampus; IH, surgical ipsilateral hippocampus; KA, kainic acid; NS, normal saline. Shapiro–Wilk test is applied for normality checks. *T*‐test is used for two groups or ANOVA for multiple groups if data are normal; otherwise, we used Wilcoxon test for non‐normal data.

To further investigate the function of *Plaur* and whether inhibition of its expression could exert a neuroprotective effect, we relied on AAV to achieve specific downregulation of *plaur* in the hippocampal region of mice (Figure 6A). Notably, 28 d after AAV injection, the virus was only distributed in this half of the brain region where AAV was injected (Figure 6B). The results of the study showed that AAV‐*Plaur* significantly downregulated the mRNA expression level of *Plaur* in the hippocampal region of mice (Figure 6C).

Encouragingly, AAV‐*Plaur* significantly suppressed KA‐induced seizures in mice (Figure 6D–F). Compared with the mice in the epileptic group, mice in the AAV‐*Plaur* group were effectively controlled in terms of both seizure level, duration of single seizure, and number of seizures in 30 min. We also found that AAV‐*Plaur* significantly inhibited KA‐induced neuronal loss in the CA1 and CA3 regions of the hippocampus in mice (Figure 6G,H). In addition, the mRNA expression of *Acsl4*, *Gpx4*, *Ptgs2*, and *Slc7a11* did not differ significantly between the groups (Figure 6I).

## 4 DISCUSSION

**FIGURE 7:**
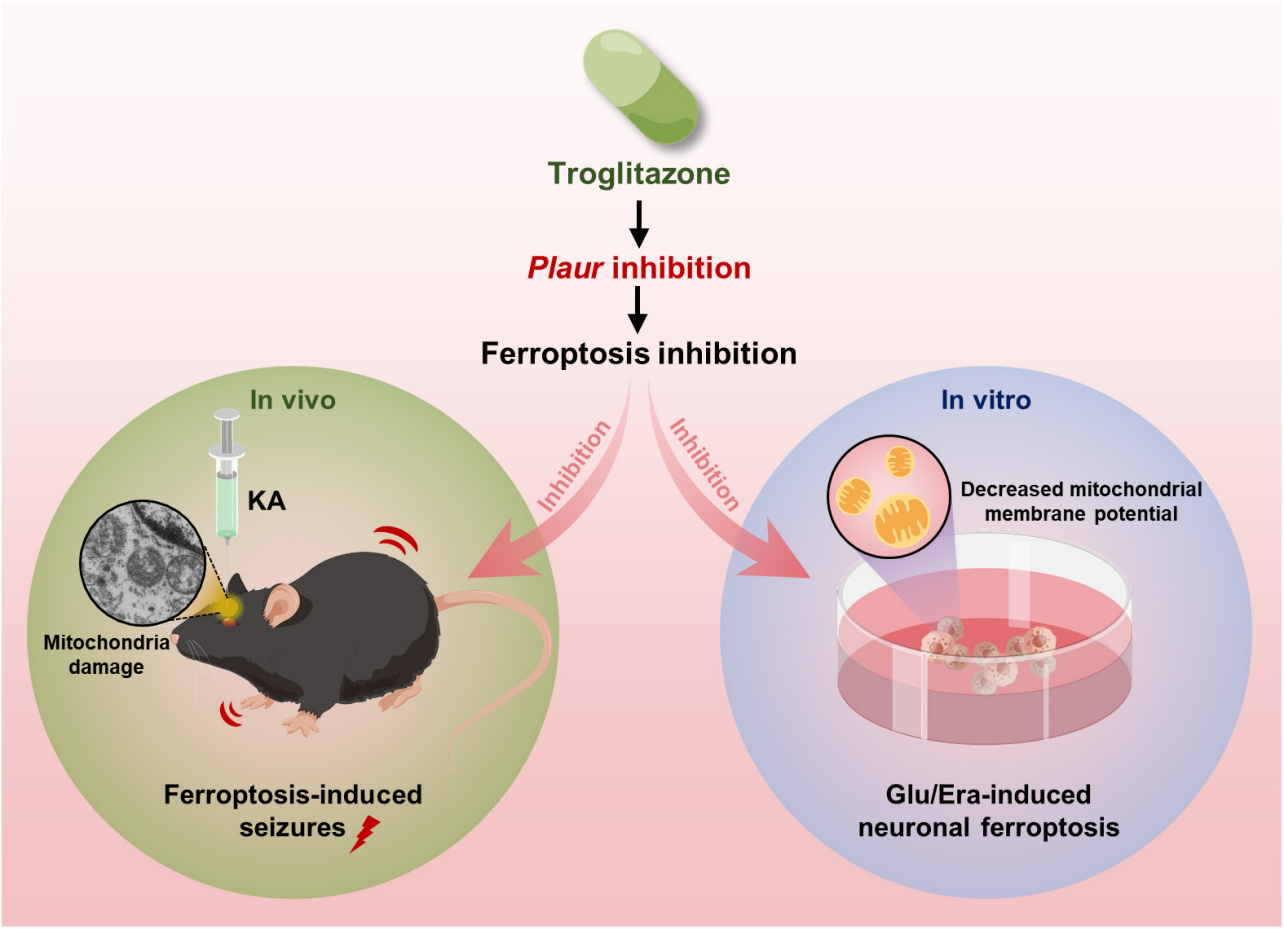
Working model summarizes the protective effect of troglitazone on KA‐induced seizures and neuronal damage. Era, erastin; Glu, glutamate; KA, kainic acid. Drawn by Figdraw (www.figdraw.com)

Using the HT22 hippocampal neuronal model and the KA‐induced mouse epilepsy model, we found that TGZ could ameliorate neuronal damage in HT22 cells and KA‐induced seizures in mice, and inhibit the alteration of mitochondrial morphology in neuronal cells of the epileptic mouse brain; RNA‐seq sequencing revealed that TGZ may exert neuroprotective effects through regulating *Hbegf* and *Plaur*; using AAV to downregulate *Plaur* expression in the hippocampal region of epileptic mice can alleviate seizures and attenuate neuronal damage in the hippocampal region, providing a reference for finding clinical treatments for epileptic diseases (Figure 7). A previous study demonstrated that TGZ is capable of inhibiting erastin‐induced ferroptosis in SK‐HEP‐1 liver cancer cells,^33^ which was in part consistent with our results shown in HT22 neuronal cells.

System Xc^−^ is a glutamate and cystine retrotransporter that is widely distributed on the surface of neuronal membranes and transports cystine intracellularly while expelling glutamine extracellularly. The intracellularly transferred cystine is then synthesized into glutathione in an enzymatic reaction.^34^, ^35^, ^36^ SLC7A11 is a subunit of system Xc^−^, and P53 can play an important role in reducing glutathione and thus accumulating ROS and triggering cell damage by inhibiting SLC7A11.^37^, ^38^ SLC7A11 protects cells from ferroptosis injury, and in our results (Figure 4J), *Slc7a11* expression was significantly elevated in both the glutamate‐treated and erastin‐treated groups, suggesting that *Slc7a11* may stressfully undergo a compensatory effect and thus more proactively resist ferroptosis. In contrast, when TGZ was applied to HT22 cells alone, no increase in *Slc7a11* expression was observed. This suggests that it is possible that the elimination of oxidative damage induced by glutamate or erastin by TGZ does not act through *Slc7a11*.

Both erastin and RSL3 induce ferroptosis by a mechanism that affects GPX4, with the former inactivating GPX4 indirectly by affecting cystine uptake and the latter inactivating GPX4 directly.^17^, ^39^, ^40^ GPX4 is a potent antioxidant in cells and System Xc^−^ can promote the antioxidant effects of GPX4. In our results (Figure 4J), the erastin‐treated group showed increased levels of *Gpx4* expression, while the glutamate‐treated group did not show an increase in *Gpx4*. Since we were trying to find a regulatory mechanism that is common to both glutamate and erastin models of injury, it is suggested that this mode of regulation does not act through modulation of *Gpx4*. In summary, it is likely that there is a pathway that inhibits ferroptosis independent of *Slc7a11* and *Gpx4* during the neuroprotective effects of TGZ.

*Acsl4* is a key gene in the process of ferroptosis, and *Acsl4* activates long‐chain polyunsaturated fatty acids to participate in the synthesis of membrane phospholipids.^41^ In the presence of factors such as the ferroptosis inducer RSL3, these long‐chain polyunsaturated fatty acids on membranes can often be oxidized, which in turn triggers the appearance of ferroptosis.^42^, ^43^, ^44^, ^45^ This suggests that if *Acsl4* can be inhibited, it may be possible to protect cells from ferroptosis. The inhibitory effects of TGZ, rosiglitazone, and pioglitazone on ferroptosis have been confirmed, primarily through the suppression of ACSL4.^46^ Reports suggest that TGZ can effectively suppress lipid peroxidation in tissue damage induced by intestinal injury by inhibiting ACSL4.^47^ In our experiments (Figure 4J), it was found that *Acsl4* expression levels were significantly reduced in HT22 cells in the glutamate‐treated group, while the TGZ combined with glutamate group showed significantly higher *Acsl4* expression levels compared with the glutamate‐treated group. This is in contrast to the inhibition of *Acsl4*‐induced ferroptosis, suggesting that the cell damage in the glutamate model was not due to the classical *Acsl4* inhibition mode and that the neuroprotective effect of TGZ was not mediated by the regulation of *Acsl4*, especially in the erastin‐modified group. In particular, *Acsl4* expression did not change in the erastin‐modified group. That is, TGZ may regulate more genes in ferroptosis than *Acsl4*.

It is well known that thiazolidinediones, such as TGZ, can increase insulin sensitivity and enhance glucose metabolism and ultimately lower glucose by targeting and activating PPAR‐γ in the nucleus of cells. So, is the inhibitory effect of TGZ on ferroptosis related to its activation of PPAR‐γ? In recent years, an increasing number of researchers have highlighted the potential involvement of PPARγ in the control of epileptic seizures.^48^ One study found that the use of thiazolidinediones such as TGZ inhibited RSL3‐induced ferroptosis, but the use of non‐thiazolidinedione PPAR‐γ agonists such as GW1929 did not inhibit RSL3‐induced ferroptosis.^27^ This suggests that the inhibitory effect of TGZ on ferroptosis may not be exerted through activation of PPAR‐γ. However, on the contrary, a study conducted by Wang et al. suggests that activation of PPARγ in rats can inhibit hippocampal neuronal damage caused by ferroptosis.^49^ This inconsistency may arise from variations in the methods used to create different epilepsy models. Therefore, it becomes essential to perform a comparative analysis of the protective effects and mechanisms of TGZ in different epilepsy models.

Currently Rezulin (TGZ) is available in the USA. TGZ is well absorbed orally, reaching maximum blood concentration in about 2–3 h, with a half‐life of about 9 h. As a hypoglycemic agent, the daily dose of 200–600 mg is usually taken once daily with breakfast. Considering clinical reports that TGZ is associated with atopic hepatotoxicity, there are very few cases of liver failure, liver transplantation, or death in its post‐marketing clinical use.^28^, ^50^, ^51^ Therefore, its safe use in doses is particularly important. The US FDA recommends that liver function be tested when using thiazolidinediones for glucose‐lowering therapy, and that basal alanine aminotransferase (ALT) levels should not exceed 2.5 times the upper limit of normal before treatment, be tested every 2 months for 1 year after the start of treatment, and be discontinued if ALT levels exceed three times the upper limit of normal.^52^ The doses of TGZ administered in this study were 5 mg/kg/day versus 20 mg/kg/day. In previous studies, no significant hepatic impairment such as diffuse hepatic necrosis was observed when TGZ was administered to male mice at 400 mg/kg or to female mice at 800 mg/kg.^53^

**FIGURE 8:**
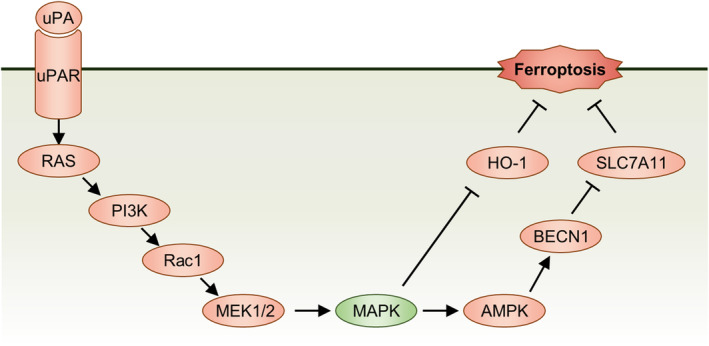
In the process of neuronal resistance to ferroptosis, Plaur may influence upstream regulators of ferroptosis such as HO‐1 and SLC7A11 through the RAS/MAPK pathway, thereby affecting ferroptosis. uPA, Plau; uPAR, Plaur.

The epidermal growth factor receptor (EGFR) is a tyrosine kinase type receptor that activates downstream signaling pathways such as MAPK/ERK and PI3K/AKT pathways by binding to ligands such as EGF/HBEGF. In the pathway analysis of Figure 5, the MAPK pathway was enriched (the most significant). The c‐Jun N‐terminal kinase (JNK) and p38 MAPK within the MAPK pathway are considered to be related to the induction of ferroptosis. In addition, the classic MAPK signaling pathway of RAS–RAF–MEK–ERK can regulate the intake and storage of iron ions, affecting the metabolic pathways of iron ions, thereby influencing the occurrence of ferroptosis. This suggests that troglitazone may use *Plaur* as a regulatory gene to achieve the ultimate result of inhibiting ferroptosis by affecting the MAPK pathway (Figure 8).

Previous studies have shown that *Plaur* expression is altered in the hippocampus during epilepsy‐related circuit reorganization. In the normal hippocampus, *Plaur* expression is low and limited to a small number of astrocytes and interneurons. In animals with seizures, *Plaur* expression was significantly increased, peaking at 1 and 4 days after the seizure. Immunohistochemical double‐labeling showed that *Plaur* was highly expressed in neurons of the hippocampus and dentate gyrus. The authors concluded that the increased expression of *Plaur* in the post‐neural injury phase suggests that it plays an active role in brain tissue remodeling and recovery of brain function. In our study, the same high expression of *Plaur* was found in the epileptic foci (right hippocampus in mice).

It has been noted that NMDA receptor signaling was found to be significantly downregulated in mice with *Hbegf* deletion in hippocampal regions.^54^, ^55^ Whereas NMDA receptors are specific excitatory glutamate receptors that play an important role in epileptogenesis, NMDA receptor antagonists attenuate epilepsy‐induced neuronal loss in the hippocampal CA1 and CA3 regions in mice. This suggests that in our experimental results, the downregulation of *Plaur* exerts an anti‐epileptic effect, possibly through the inhibition of NMDA receptor signaling.

In summary, we report that TGZ protects against glutamate‐ and erastin‐induced neuronal cell injury, downregulates Lipid‐ROS induced by glutamate and erastin, and restores the decreased membrane potential of neuronal cell mitochondria caused by glutamate and erastin. TGZ ameliorated seizures and neuronal damage induced by KA in mice, and inhibited mitochondrial morphology changes in neuronal cells of epileptic mice. The use of AAV‐*Plaur* to downregulate *Plaur* expression levels in the hippocampal region of epileptic mice alleviated seizures and attenuated neuronal damage in the hippocampal region, suggesting that *Plaur* may be a regulatory gene for the anti‐epileptic neurological damage effect of TGZ, providing a reference for the search of clinical treatments for epileptic diseases.

The results of this study can provide experimental and theoretical basis for the use of troglitazone in the treatment of epilepsy and also offer new ideas and strategies for reducing the toxic side effects of commonly used anti‐epileptic drugs and alleviating drug‐resistant epilepsy in clinical practice. In addition, there are still deficiencies in this study, such as how troglitazone regulates iron metabolism and ferroptosis, which requires further in‐depth investigation at the mechanistic level.

## AUTHOR CONTRIBUTIONS

ZBW conducted the experiments and wrote the manuscript. JYL, SLJ, and WZ gave help substantially in the experiment. JYL and PX gave help in the data analysis. ZQL, XYM, and WTD revised the manuscript. ZQL approved the final manuscript. ZQL and XYM contributed equally to this article.

## FUNDING INFORMATION

This work was supported by the National Natural Science Foundation of China (82173901), Major Project of Natural Science Foundation of Hunan Province (Open competition, 2021 JC0002), Major Science and Technology Program of Changsha (kh2003010), Hunan Provincial Natural Science Foundation of China (2023JJ40415), and Changsha Municipal Natural Science Foundation (kq2208155), and China Postdoctoral Science Foundation (2023M741145).

## CONFLICT OF INTEREST STATEMENT

The authors declare that they have no potential conflict of interest.

## Supporting information


Data S1.

## Data Availability

The data that support the findings of this study are available from the corresponding author upon reasonable request.
