# Supplementary figures and images for: Unveiling the shield: Troglitazone's impact on epilepsy‐induced nerve injury through ferroptosis inhibition
Source: CNS Neurosci Ther. 2024 Aug 15;30(8):e14911. doi: 10.1111/cns.14911 (PMC11325165; doi:10.1111/cns.14911)

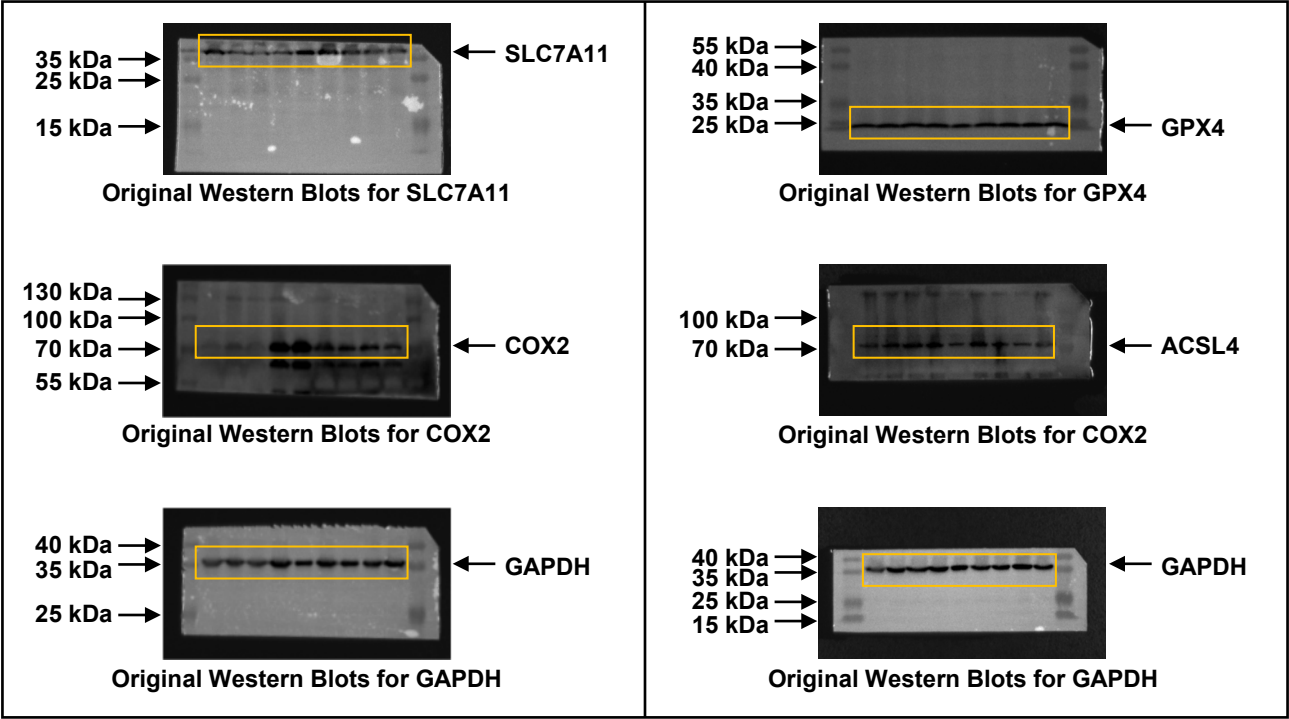

Full unedited gel/blot for Figure 2E.

Supplement: Supplementary file 1 — Data S1. [file CNS-30-e14911-s001.pdf]
